# Anatomical variations along the leaf axis modulate photosynthetic responses of sorghum and maize under different water availabilities

**DOI:** 10.1111/plb.70084

**Published:** 2025-07-30

**Authors:** J. P. V. de Oliveira, V. P. Duarte, C. H. G. dos Reis, P. N. da Silva, E. M. de Castro, P. C. Magalhães, F. J. Pereira

**Affiliations:** ^1^ Universidade Federal de Lavras, Campus Universitário Lavras Minas Gerais Brazil; ^2^ Embrapa Milho e Sorgo de Sete Lagoas Sete Lagoas Minas Gerais Brazil; ^3^ Instituto de Ciências da Natureza (ICN) Universidade Federal de Alfenas Alfenas Minas Gerais Brazil

**Keywords:** Chlorophyll fluorescence, gas exchange, leaf parts, mesophyll, water relations

## Abstract

- Water limitation leads to alterations in plants, including tolerance responses. Maize and sorghum are both C4 crops with contrasting drought tolerance, where several aspects of leaf anatomy and physiology are unclear. This work aimed to investigate the effect of drought on anatomical and photosynthetic traits along the leaf axis of maize and sorghum.
- Experiments were conducted in a greenhouse with maize and sorghum exposed to three irrigation conditions (field capacity (FC), 75% FC, and 50% FC). Three leaf regions (base, middle, and tip) were assessed for photosynthetic and anatomical parameters.
- Water limitation promoted reductions in maize leaves in terms of water‐use efficiency, leaf thickness, xylem vessel diameter, and area of the bundle sheath; however, sorghum leaves increased these under the same conditions. The middle region of the leaf had higher values than other leaf parts for most parameters, while sorghum had increased ΦPSII, Fv/Fm, and ETR at the leaf base. Photochemical values increased in both species under water limitation. Maize had increased stomatal density compared to sorghum, which led to higher transpiration rates.
- Anatomical and photosynthetic traits varied along the leaf axis and were more reduced in maize than sorghum under drought. The middle region of the leaf was most responsive to these changes in both species.

## Graphical abstract

The middle part of C_4_ leaves is more anatomically and photochemically drought‐adapted than the apex or base. Water limitation did not negatively effect leaf anatomy and photosynthesis in sorghum, but reduced gas exchange and the thickness of leaf tissues in maize.

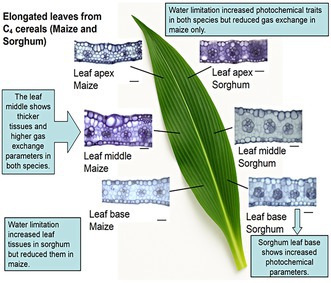

## INTRODUCTION

Climate changes have led to more frequent water limitation events, thus reducing plant productivity (McDowell *et al*. 2022). Water limitation severely impacts crop production, with agricultural losses up to US$30 billion (Gupta *et al*. 2020). Hence, drought events have become a global concern (Kim *et al*. 2019; Zafar *et al*. 2023).

Drought promotes water stress in plants leading to several modifications to anatomical and physiological traits in leaves. According to Munné‐Bosch & Villadangos (2023), water limitation reduces vegetative growth, chlorophyll fluorescence, photosynthesis, and gas exchange because of stomatal limitation. Thus, water limitation is a major factor reducing crop production and food safety (Rehman *et al*. 2023), leading to the need to find tolerant crops and understand crop tolerance mechanisms.

*Zea mays* L. is a high water‐demanding and drought‐sensitive species (de Oliveira *et al*. 2023; Shahzad *et al*. 2023); while *Sorghum bicolor* (L.) Moench. is a more drought‐tolerant and efficient C4 plant (Impa *et al*. 2019; de Oliveira *et al*. 2023). Sorghum is often used as a model for the study of water limitation (Yang *et al*. 2020). Water limitation reduces leaf area, leaf thickness, dry mass, and gas exchange in both maize and sorghum, but these changes are more intense in maize, while sorghum has higher stomatal control, which helps to reduce water loss (de Oliveira *et al*. 2023).

Even under water limitation, maize has a higher leaf area compared to sorghum, which is one of the main limiting factors under drought conditions (de Oliveira *et al*. 2023). Plant growth shows spatial variation related to drought tolerance because of a correlation between axis morphology and functional traits (Ford *et al*. 2008; Normand *et al*. 2009). Maize and sorghum are both C4 species, with long lanceolate leaves, but variations in leaf anatomical and physiological characteristics are often overlooked in drought studies of such crops.

Maize and sorghum both have Kranz anatomy (de Oliveira *et al*. 2023) and C4 metabolism (Weber & von Caemmerer 2010) but differ in leaf size and drought tolerance (de Oliveira *et al*. 2023). Both species originate from dry and hot environments, with high radiation (Sage 2004). C4 species with Kranz anatomy show spatial separation in photosynthetic tissues, with phosphoenolpyruvate carboxylase (PEPCase) in mesophyll cells that produces a C4 acid which is then transported to bundle sheath cells containing Rubisco, where the Calvin‐Benson cycle is located (Sage 2004; Weber & von Caemmerer 2010). Water stress causes biochemical and anatomical limitations, reducing photosynthesis (de Oliveira *et al*. 2023; Zahra *et al*. 2023). Anatomical variations along the leaf axis of both maize and sorghum is little understood and may influence photosynthesis.

Generally, the middle part of the leaf is used in anatomical and physiological studies (Taratima *et al*. 2020; Della Torre *et al*. 2021); however, few studies have evaluated the anatomy, gas exchange, or chlorophyll fluorescence in different leaf parts. It is known that leaf anatomy changes along the leaf axis, such as the shape and amount of vascular tissue (Gavilanes *et al*. 2016), stomatal density (Hsie *et al*. 2015; Mansoor *et al*. 2019), stomatal index (Silva *et al*. 2009; Della Torre *et al*. 2021), and mesophyll thickness (Wyka *et al*. 2019; Della Torre *et al*. 2021; de Oliveira *et al*. 2022). Thus, such anatomical and physiological traits are expected to vary along the leaf axis. Because photosynthesis is spatially separated between mesophyll and bundle sheath cells in C4 plants (Weber & von Caemmerer 2010), anatomical evaluation of photosynthetic tissues must also include bundle sheath traits; however, these cells are generally neglected in leaf anatomical analysis of maize and sorghum under drought.

The hypotheses of this study were: (1) anatomical variations in photosynthetic tissues and stomatal traits along the leaf axis of maize and sorghum affect gas exchange and photochemical responses. (2) Water limitation modifies specific leaf parts which may improve drought tolerance in maize and sorghum. Thus, this work aimed to evaluate drought effects on the anatomy, gas exchange, and photochemical responses at the base, apex, and middle regions of maize and sorghum leaves.

## MATERIAL AND METHODS

### Plant material and experimental design

The experiment was carried out in a greenhouse at the Universidade Federal de Lavras (UFLA), state of Minas Gerais, Brazil (21°13′17″S, 44°57′47″W). *Sorghum bicolor* (L.) Moench and *Zea mays* L. were obtained from seeds provided by the Embrapa National Research Center for maize and sorghum, Minas Gerais, Sete Lagoas, Brazil.

Seeds were sown in 5.0 L plastic pots containing 2.0 L sand and 800 ml nutrient solution (Hoagland & Arnon 1950) at 40% concentration. Pots were then placed in a germination chamber under constant light at 25 °C for 7 days, after which seedlings had three leaves and were 10‐cm tall. Seedlings of similar size were individually transplanted into 5.0 L plastic pots containing 3.0 L sand. These pots were kept in a greenhouse at 25 °C ± 2 °C, 50% relative humidity, and a 12 h photoperiod, with average PAR of 730 μmol m^−2^ s^−1^. Experiments using maize or sorghum were conducted side by side in the greenhouse, exposing the plants to the same conditions.

The plants were then subjected to three water conditions: These were (1) field capacity (FC), (2) 75% field capacity (75% FC), and (3) 50% field capacity (50% FC). Field capacity was considered as the maximum volume of water retained by 1.0 L of sand without becoming waterlogged. The volume of water applied to achieve FC was 310.0 ml L^−1^ sand, and for 75% FC and 50% FC, the amount of water in the substrate were maintained at 232.5 and 155.0 ml L^−1^ sand, respectively. The first irrigation used 40% nutrient solution (Hoagland & Arnon 1950), then the water lost by evapotranspiration was monitored as the daily difference in mass of each pot. Water was replaced daily and the nutrient solution replaced weekly for 60 days. The experiment was completely randomized, with three treatments and ten replicates (*n* = 30), each replicate comprised one plant per pot. All data obtained in multiple assessments (e.g., two leaves per plant in gas exchange) were then averaged for each replicate (maintaining *n* = 30 and avoiding artificial replication).

### Gas exchange and chlorophyll content

The gas exchange was assessed on three leaf regions (apex, middle, and base) using an infrared gas analyser model LI‐6400XT (LI‐COR Biosciences, Lincoln, USA) coupled to an LI‐6400‐02B cuvette, and a red/blue LED source (LI‐COR). Measurements were taken from two fully developed leaves per plant/replicate between 08:00 h and 10:00 h, with photon flux density fixed at 1000 μmol m^−2^ s^−1^, and mean temperature of 28.1 °C. Atmospheric CO_2_ concentration was 420.1 μmol mol^−1^, and pump flow rate was 500 μmol s^−1^. Net photosynthesis (A) and transpiration (E) were assessed. Chlorophyll content was assessed using a chlorophyll meter, SPAD‐502 (Konica‐Minolta, Japan) in the leaf base, middle, and apex. At the end of the experiments, instantaneous water‐use efficiency (WUE) was calculated as the ratio of net photosynthesis (A) to transpiration (E) (A/E).

### Chlorophyll fluorescence analysis

The chlorophyll fluorescence was assessed with a portable modulated fluorometer MINI‐PAM (Heinz Walz, Effeltrich, Germany). Maximum photochemical yield of PSII (Fv/Fm), electron transport rate (ETR), and effective photochemical yield of PSII (ΦPSII) were assessed. Measurements in darkness were performed using aluminium clips (DLC‐8) coupled to the fibre optic cable of the MINI‐PAM at all three leaf regions (apex, middle, and base) of two fully expanded leaves per plant/replicate. Dark evaluations were taken after at least 30 min acclimation. Calculations for ΦPSII, Fv/Fm, and ETR were performed according to Mini‐Pam II user manual (Heinz Walz 2022). Fv/Fm was calculated as: Fv/Fm = [(Fm)max – (F_0_)max]/(Fm)max, where (Fm)max is maximum Fm and Fm is maximum fluorescence elicited by a pulse of saturating light (Saturation pulse) which closes all photosystem II reaction centers, (F_0_)max is maximum F_0_, which is the minimum fluorescence excited by very low intensity measuring light to maintain photosystem II reaction centers open. ΦPSII was calculated as: ΦPSII = (Fm′ – F)/Fm′, where Fm′ is maximum fluorescence of an illuminated sample, and F is momentary fluorescence of an illuminated sample shortly before application of a saturated light pulse. ETR is calculated as: ETR (II) = PAR × ETR‐Factor × (PPS2/PPS1 + 2) × ΦPSII, where PAR is quantum flux density of photosynthetically active radiation (PAR) impinging on the sample, ETR‐Factor is sample absorptance (= 1−transmittance), and PPS2/PPS1 + 2 is relative distribution of absorbed PAR to photosystem II.

### Anatomical analysis

Two fully developed leaves per plant/replicate were removed and fixed in 70% ethanol (Johansen 1940). Sampled leaves were those evaluated in gas exchange, photochemical, and chlorophyll measurements. Transverse sections were performed using steel blades at the apex, middle, and base leaf regions. The sections were clarified with 50% sodium hypochlorite and washed twice in distilled water for 10 min. Further, sections were stained with safrablau solution (1% safranine and 0.1% astra blue at 1:7) then mounted on slides with 50% glycerol (Johansen 1940). Paradermal imprints (Segatto *et al*. 2004) were taken from both abaxial and adaxial leaf sides at the apex, middle, and leaf base. These imprints were obtained by covering the leaf surface with a thin layer of cyanoacrylate resin, which was removed after polymerization then mounted on slides with coverslips. The paradermal imprints were taken in the morning from 06:00–08:00 h when stomata were expected to be open. The slides were observed and images taken under a CX31 light microscope (Olympus, Tokyo, Japan). One slide per leaf was made and, three sections and four fields were analysed for each slide, and data were averaged for each replicate. The images were analysed with ImageJ software v. 1.45 s (Wayne Rasband National Institutes of Health, USA).

The anatomical characteristics of transverse sections assessed were: leaf thickness, proportion of mesophyll cells, bundle sheath area, proportion of vascular tissue, and xylem vessel diameter. Mesophyll thickness and xylem vessel diameter were measured directly using Image J software. For proportion of mesophyll, bundle sheath area, and vascular tissues we used Image J to measure area of these tissues. The proportion (P%) was calculated as: P% = (area of tissue/total area of leaf section) × 100. For paradermal sections, the following structures were analysed: adaxial and abaxial stomatal index and abaxial and adaxial stomatal density (SD). SD was calculated as: SD = number of stomata × (10^6^/section area).

### Statistical analysis

The data were subjected to two‐way ANOVA, and means were compared using the Scott‐Knott test at *P* < 0.05 using SISVAR v. 5.0 (Ferreira 2011). Before parametric analysis, data were tested for normality using the Shapiro–Wilk test, and all variables showed normal distribution.

## RESULTS

**Fig. 1:**
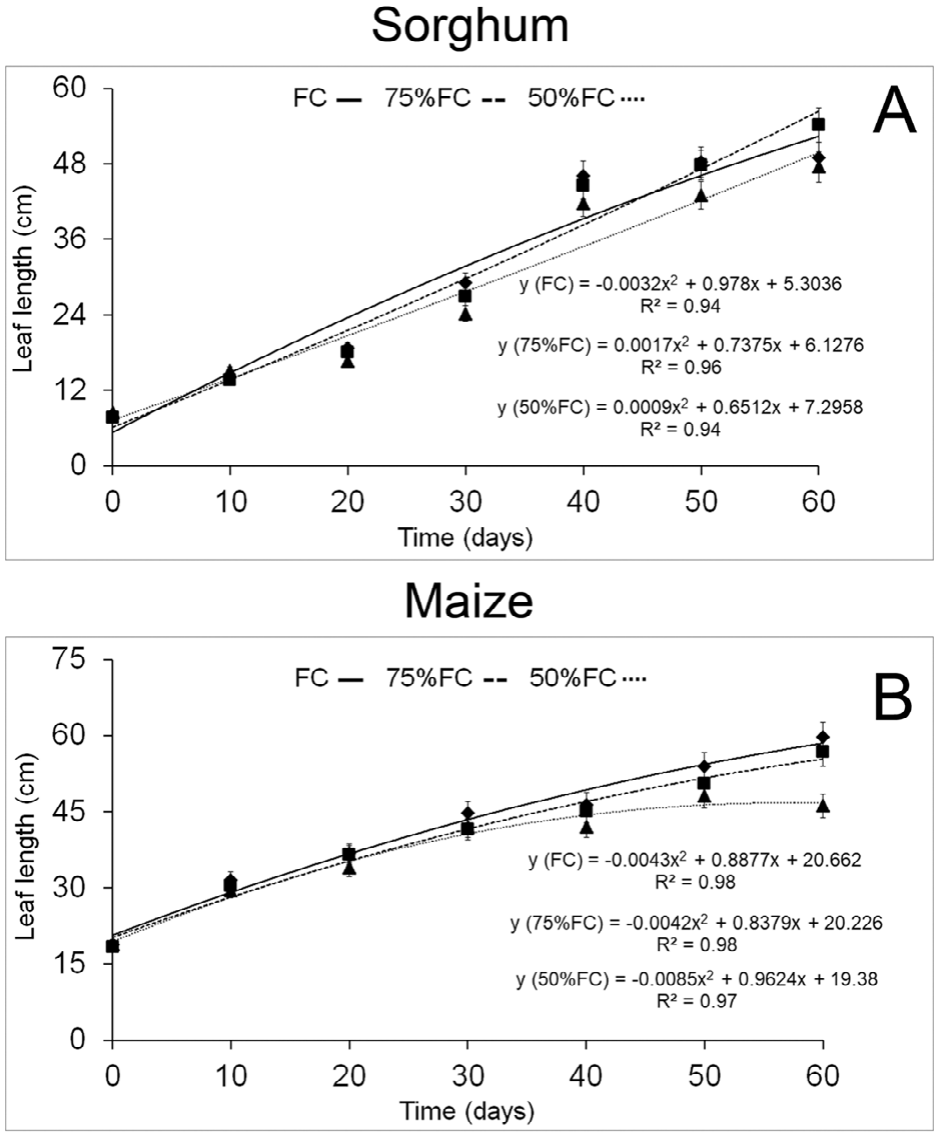
Leaf length of *Zea mays* and *Sorghum bicolor* under field capacity (FC), 75% FC, and 50% FC. Bars = standard error (*n* = 30).

The ANOVA data for maize and sorghum are provided in the Data S1. Both maize and sorghum leaves increased in length during the experimental period (Fig. 1). Sorghum leaves showed similar elongation under all water conditions, but 75% FC surpassed other treatments at the end of the experiment (Fig. 1A). Nonetheless, water limitation under 50% FC significantly reduced leaf length of maize after 40 days of the experiment (Fig. 1B).

**Fig. 2:**
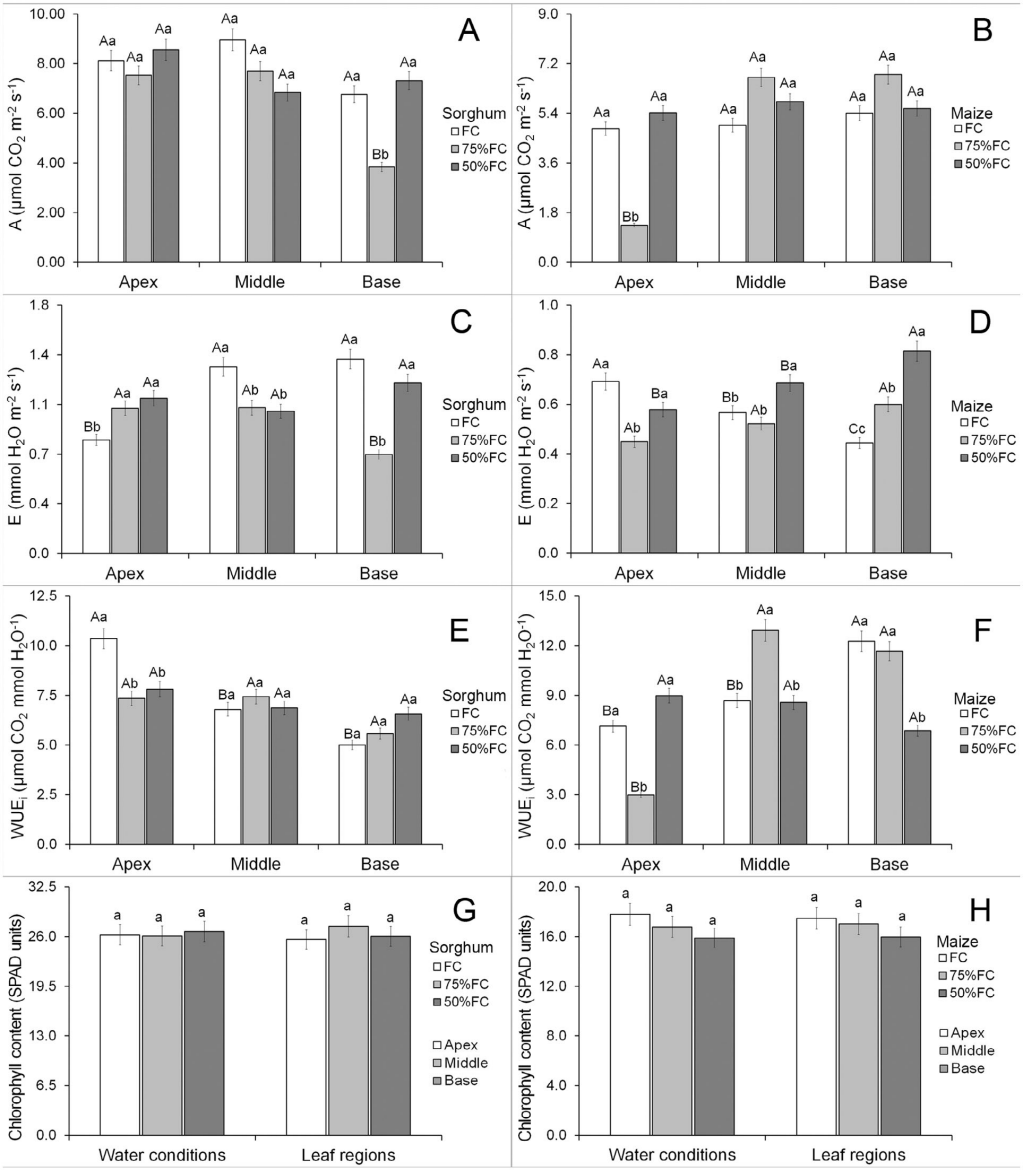
Leaf gas exchange parameters and chlorophyll content of *Sorghum bicolor* and *Zea mays* under different water conditions. A, Net photosynthesis; E, Transpiration; WUEi, instantaneous water‐use efficiency. Lowercase letters compare water conditions and uppercase letters compare leaf regions. The same letter denotes no significant differences according to the Scott–Knott test at *P < 0.05*. Bars = standard errors (*n* = 30).

There was a positive interaction for all gas exchange and chlorophyll content parameters (Data S1). Water limitation did not modify photosynthesis in the leaf apex and middle regions of sorghum leaves, but 75% FC reduced this parameter in the leaf base (Fig. 2A). Photosynthesis of sorghum was similar in different leaf parts under FC and 50% FC, but lower in the leaf base under 75% FC (Fig. 2A). Photosynthesis in maize was similar under FC and 50% FC; however, it was reduced under 75% FC in the leaf apex (Fig. 2B). Photosynthesis of leaf middle and base parts were similar under FC and 50% FC, but lower in the leaf apex of maize under 75% FC (Fig. 2B).

Water limitation increased transpiration in the apex of sorghum leaves but had no effect on the middle part, while 75% FC reduced transpiration in the leaf base (Fig. 2C). Transpiration in sorghum was lower in the leaf apex under FC, and even lower at the leaf base under 75% FC, while mean values were similar in all leaf parts under 50% FC (Fig. 2C). Drought reduced transpiration in maize leaves only in the apex at 75% FC; however, transpiration increased under 50% FC in the middle region, and by 50% at 75% FC in the leaf base (Fig. 2D).

Drought reduced water‐use efficiency in the apex of sorghum leaves, but middle and base parts remained unaffected (Fig. 2E). The apex of sorghum leaves had higher water‐use efficiency under FC, but there were no significant differences in water limitation (Fig. 2E). Drought reduced water‐use efficiency of maize leaves in the apex only under 75% FC, and at the base under 50% FC; however, 75% FC increased this parameter in the middle leaf part (Fig. 2F). Under FC, water‐use efficiency of maize leaves was higher in the base, but 75% FC increased this in both leaf base and middle parts, while there were no significant differences in this parameter at 50% FC (Fig. 2F).

**Fig. 3:**
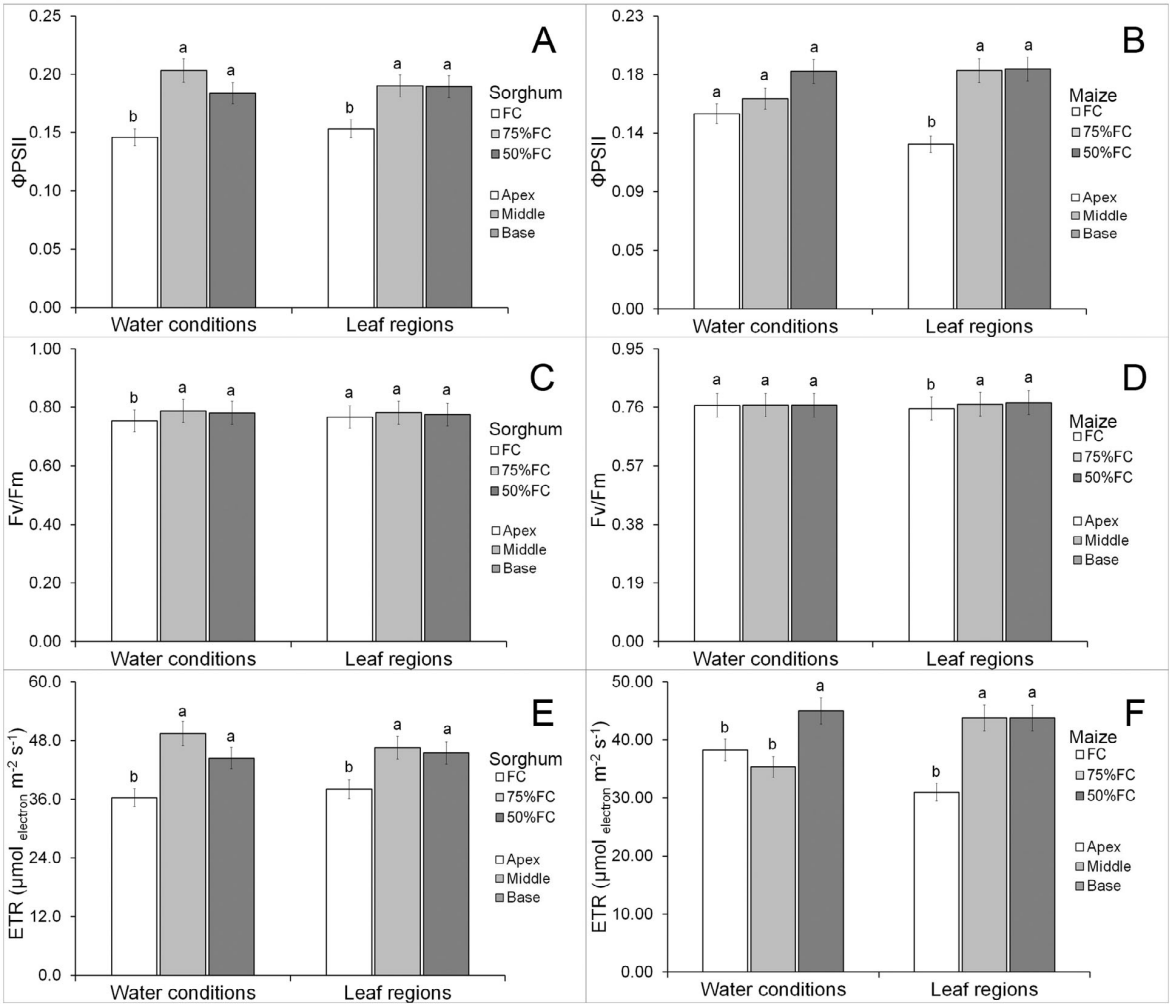
Chlorophyll fluorescence parameters of *Sorghum bicolor* and *Zea mays* under different water conditions. ΦPSII, photochemical efficiency of PSII; ETR, electron transport rate; Fv/Fm, maximum PSII quantum yield. The same letter denotes no significant differences according to the Scott–Knott test at *P* < 0.05. Bars = standard errors (*n* = 30).

There were no effects of water limitation on chlorophyll content (Data S1) in any leaf parts of maize and sorghum leaves (Fig. 2G,H). Photochemical parameters did not show a significant interaction between leaf part and water availability (Data S1). Water limitation increased effective photochemical yield of PSII (ΦPSII) in sorghum leaves but this was lower in the apex (Fig. 3A). Drought had no significant effects on ΦPSII of maize but was lower in the leaf apex (Fig. 3B). Water limitation increased maximum photochemical yield of PSII (Fv/Fm) in sorghum leaves, but there was no significant difference between leaf parts (Fig. 3C). Drought did not promote significant modifications in Fv/Fm in maize leaves but the apex had lower means compared with the other parts (Fig. 3D). Drought increased electron transport rate (ETR) in sorghum leaves, but this was lower in the leaf apex (Fig. 3E). The 50% FC increased ETR in maize leaves but was lower in the leaf apex (Fig. 3F).

**Fig. 4:**
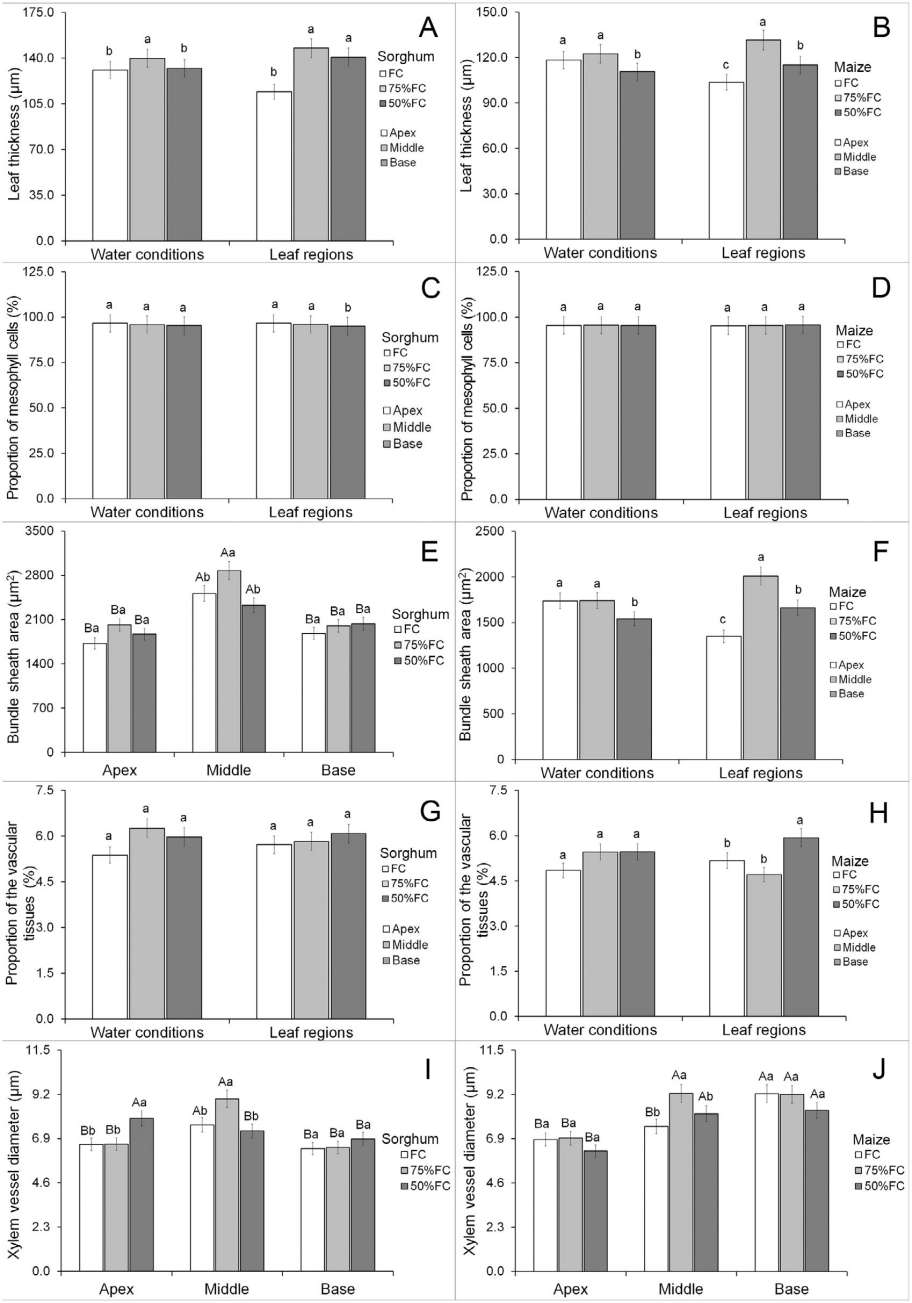
Leaf anatomical characteristics of *Sorghum bicolor* and *Zea mays* under different water conditions. Lowercase letters compare water conditions, and uppercase letters compare leaf regions. The same letter denotes no significant differences according to the Scott–Knott test at *P* < 0.05. Bars = standard errors (*n* = 30).

In transverse leaf sections, only area of bundle sheath cells and xylem vessel diameter showed significant interactions (Data S1). The 75% FC treatment increased sorghum leaf thickness except in the apex compared with other parts (Figs. 4A and 6). Treatment with 50% FC reduced leaf thickness in maize, but the middle part of the leaf was thicker while the apex was thinner (Figs. 4B and 7). Water limitation did not significantly change the proportion of mesophyll cells in sorghum leaves, except at the base where there was less mesophyll tissue compared with other regions (Figs. 4C and 6). The proportion of mesophyll cells was not significantly modified by water limitation or leaf part in maize (Figs. 4D and 7).

The 75% FC treatment increased the area of bundle sheath cells in the middle region of the leaf, but did not affect other leaf parts (Figs. 4E and 6). The middle region of the sorghum leaf had larger bundle sheath cells under all water treatments compared with other leaf parts (Figs. 4E and 6). Water limitation at 50% FC reduced the size of bundle sheath cells in maize leaves (Figs. 4F and 7). Maize leaves had larger bundle sheath cells in the middle region, while the apex contained smaller cells (Figs. 4F and 7). The proportion of vascular tissue in sorghum leaves was not significantly modified by water availability or leaf region (Figs. 4G and 6). Water limitation had no significant effect on proportion of vascular tissue in maize leaves, but these tissues increased at the leaf base (Figs. 4H and 7).

Drought increased xylem vessel diameter at the sorghum leaf apex (50% FC) and middle region (75% FC) but did not affect the leaf base (Figs. 4I and 6). The middle region of sorghum leaves had larger xylem vessels under FC and 75% FC treatments and under 50% FC the leaf apex contained larger vessels (Figs. 4I and 6). The 75% FC reduced xylem vessel diameter in the middle region of maize leaves but had no effect in other leaf parts (Figs. 4J and 7). Vessel diameter was smaller at the apex of maize leaves but under FC the base region had larger vessels (Figs. 4J and 7).

**Fig. 5:**
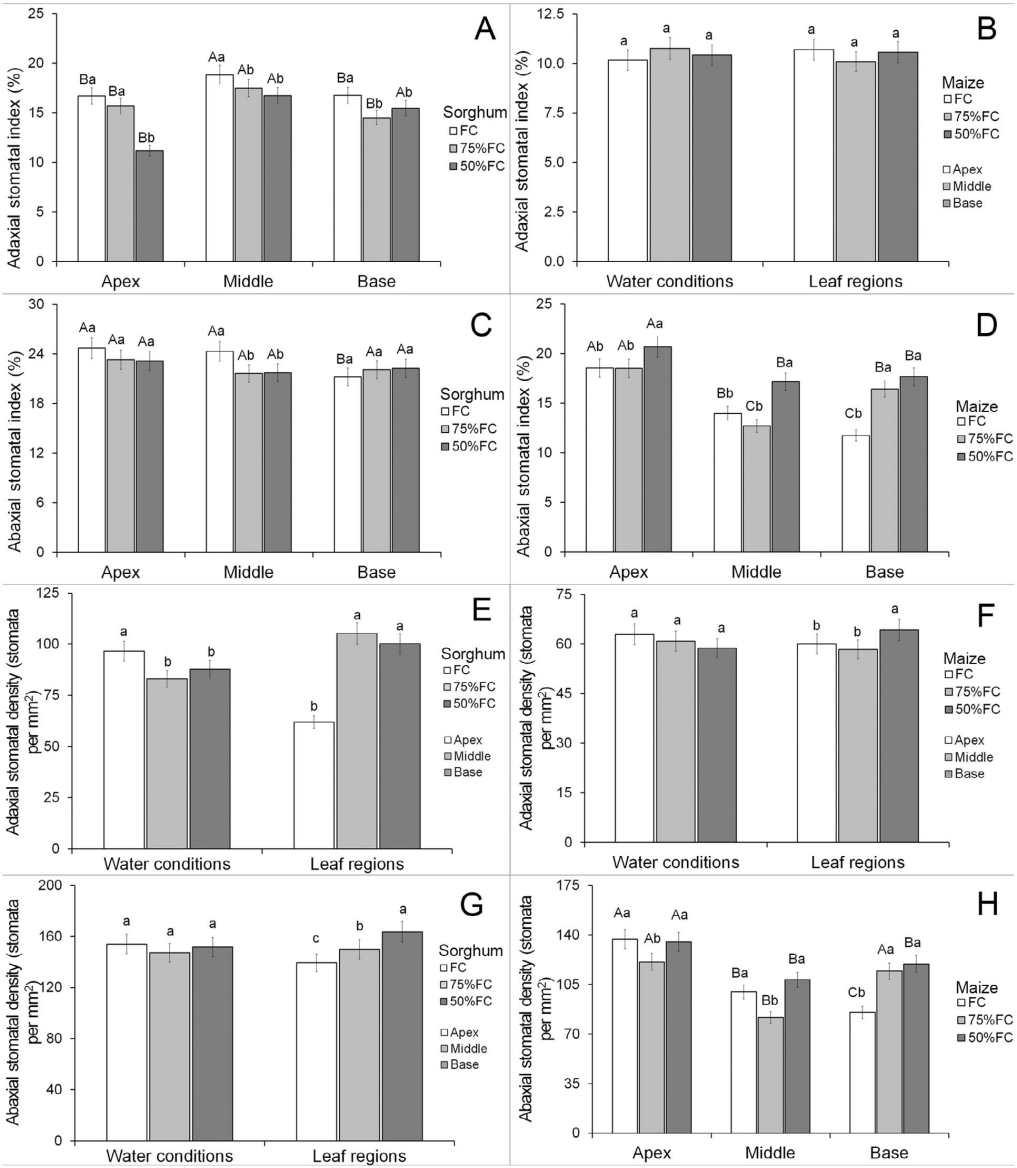
Stomatal characteristics of *Sorghum bicolor* and *Zea mays* under different water conditions. Lowercase letters compare water conditions and uppercase letters compare leaf regions. The same letter denotes no significant differences according to the Scott–Knott test at *P* < 0.05. Bars = standard errors (*n* = 30).

Data for stomatal traits showed an interaction for both adaxial and abaxial stomatal index of sorghum leaves and the abaxial stomatal index and density of maize (Data S1). Water limitation reduced the adaxial stomatal index in sorghum leaves in all three leaf regions (Fig. 5A). The middle part of sorghum leaves had a higher stomatal index, while plants under 50% FC had a lower index at the apex (Fig. 5A). The adaxial stomatal index was not significantly modified by water treatments or leaf regions in maize (Fig. 5B). Water limitation reduced the abaxial stomatal index only in the middle region of sorghum leaves (Fig. 5C). The leaf parts show little variation in the abaxial stomatal index in sorghum but this parameter was smaller at the leaf base under the FC treatment (Fig. 5C). 50% FC increased the abaxial stomatal index of all parts of maize leaves (Fig. 5D) and the leaf apex had the highest means under all irrigation conditions (Fig. 5D).

Water limitation reduced adaxial stomatal density of sorghum leaves and the leaf apex was lowest for this parameter (Fig. 5E). Adaxial stomatal density was not affected by water limitation in maize leaves, while the leaf base had higher values for this parameter (Fig. 5F). Water limitation did not promote significant modifications in abaxial stomatal density of sorghum leaves, but varied in different leaf parts, with higher means at the base and the lowest values in the apex (Fig. 5G). 50% FC reduced abaxial stomatal density in maize leaves in the apex and middle regions but increased this parameter in the leaf base (Fig. 5H). The leaf apex had higher stomatal density in maize leaves under all water conditions, except for the leaf base under 75% FC which was similar (Fig. 5H).

## DISCUSSION

Water limitation promoted several anatomical and physiological responses in maize and sorghum leaves, with some similar trends and some differences. In this work, we used water limitation sufficient to promote physiological responses without being lethal to maize and sorghum (de Oliveira *et al*. 2023). It was important to keep plants alive and to record modifications along the leaf axis within physiological norms. Severe drought can cause serious issues in leaf physiology (Munné‐Bosch & Villadangos 2023) leading to plant mortality (McDowell *et al*. 2022).

Most of the results suggest that the middle region of the leaf is most responsive in both maize and sorghum. This part appears more functional compared with the apex and base parts and had higher mean values for several important tolerance traits under water limitation. The variation along the leaf axis is a key feature of abiotic responses in plants (Li *et al*. 2020). As both maize and sorghum have lanceolate (long) leaves, this morphology creates the possibility of variations since the leaf apex is distant from the leaf base. Long maize leaves bend, causing changes in intercepted photosynthetic radiation, favouring light interception in the leaf middle part (Ford *et al*. 2008). Since drought reduces cell turgor pressure in maize (Talbi *et al*. 2020), these leaves will suffer some degree of bending under water limitation, which will affect light interception for photosynthesis and other anatomical and physiological parameters. The middle region of the leaf is the part most used in leaf anatomical and physiological studies in maize and sorghum (Cai *et al*. 2020; Chen *et al*. 2022; de Oliveira *et al*. 2022; de Oliveira *et al*. 2023). Thus, anatomical and physiological variations along the longitudinal axis of long leaves may create different microclimates that intercept radiation differently.

Under 75% FC, photosynthesis was reduced at the base of sorghum leaves whereas maize leaves showed a reduction in this parameter at the apex. This may be related in part to the lower amount of photosynthetic tissue in these regions. This is supported by the thinner apex with a lower proportion of mesophyll cells and smaller bundle sheath cells in sorghum; however, in maize, there is a smaller proportion of mesophyll and bundle sheath cells (Fig. 4). The middle region is the most functional in both maize and sorghum leaves. Monocotyledon leaves often develop short apical regions and auriculate sheaths (Ford *et al*. 2008; Paltiyan‐Bugtong *et al*. 2023). Water stress promote acclimation responses in leaves of tolerant species (López‐Hidalgo *et al*. 2023). Our results suggest that the apex and base of maize and sorghum leaves develop fewer tissues and may acclimate under water stress, generating more functional regions in the middle part of the leaf, but retaining photosynthetic activity along the leaf axis even under stress.

Transpiration was reduced with drought at the leaf apex of sorghum but further reduced in the middle part under water limitation; however, in maize, transpiration was reduced in the apex and increased in middle and base leaf parts (Fig. 2). In both species these changes suggest compensation mechanisms along the leaf, increasing transpiration in some parts while reducing it in others. This maintains the overall transpiration rate of the leaf and prevents severe damage to the leaf physiology of these species. Water limitation can lead to compensation in stomatal responses (Zafar *et al*. 2023). These changes also influence water‐use efficiency, reducing it in leaf parts where transpiration increased. Water‐use efficiency is related to stomatal responses in maize and sorghum under drought (de Oliveira *et al*. 2023). Drought reduces cell turgor in leaves (Ghaffar *et al*. 2023) and changes water‐use efficiency (Te *et al*. 2023). Transpiration changes in maize leaves reduced water‐use efficiency in the middle and base regions of the leaf. The lower radiation interception at the leaf base and apex of maize may reduce overall photosynthetic capacity; however, this does not affect sorghum, suggesting that this species has more stable middle leaf parts.

Water limitation increased photochemical traits in sorghum, which were higher at the middle and base of leaves. The absence of modifications to chlorophyll content suggests tolerance to water limitation at the levels tested. A stable chlorophyll content is often considered a drought tolerance trait in plants such as wheat (Ghaffar *et al*. 2023). The absence of negative effects of water limitation on chlorophyll content in both maize and sorghum plants is important, in part, in explaining the increased chlorophyll fluorescence parameters. Water limitation increased photochemical traits in sorghum (Fig. 3) with no significant limitation in maize. In both maize and sorghum, the middle and base leaf regions had higher photochemical parameters. This may be related to the elongated leaf morphology. Many studies use the middle region of maize and sorghum leaves for anatomical and physiological analyses (Romanowska‐Duda *et al*. 2019; Cai *et al*. 2020; de Oliveira *et al*. 2022, 2023). Our results suggest the middle and base of long leaves, like those of maize and sorghum, may have more functional traits and thus corroborate their use as sample region in previous works.

Photochemistry suggests tolerance mechanisms in sorghum under water limitation, which permit an increase of these parameters under drought; nonetheless, this response was not found in maize. This may be related to mechanisms that protect PSII under drought conditions in sorghum. Protected PSII responses were also reported by Lin *et al*. (2023) in *Erodium oxyrhinchum* M. Bieb. under drought conditions. In addition, the middle part of leaves of both species showed increased photochemical traits. Photosynthesis varies along the leaf surface (Moustakas *et al*. 2022). Drought‐tolerant species usually regulate water transport to leaves and favour the Fv/Fm parameter (Lin *et al*. 2023). Sorghum plants may increase water transport to leaves under drought and this could favour synthesis of electron acceptors from PSII. Another possibility is the capacity of sorghum to protect thylakoid membranes where PSII is found. Thylakoid membrane stability is essential for the efficient operation of PSII (Romanowska‐Duda *et al*. 2019; Stefanov *et al*. 2021). The protection of the PSII may be related to increased photochemical responses in sorghum under drought and is thus an important tolerance trait.

In this work, we evaluated an uncommon parameter that may also help explain photosynthetic responses in C_4_ species. This trait is the size (area) of bundle sheath cells. In C_4_ species Rubisco activity is exclusive to bundle sheath cells (Weber & von Caemmerer 2010; Wang *et al*. 2012), but is often overlooked in anatomical analyses. Rubisco activity is important for photochemical responses because the Calvin‐Benson cycle consumes ATP and NADPH which are the products of this photosynthesis stage, and higher consumption of such metabolites may stimulate their production.

**Fig. 6:**
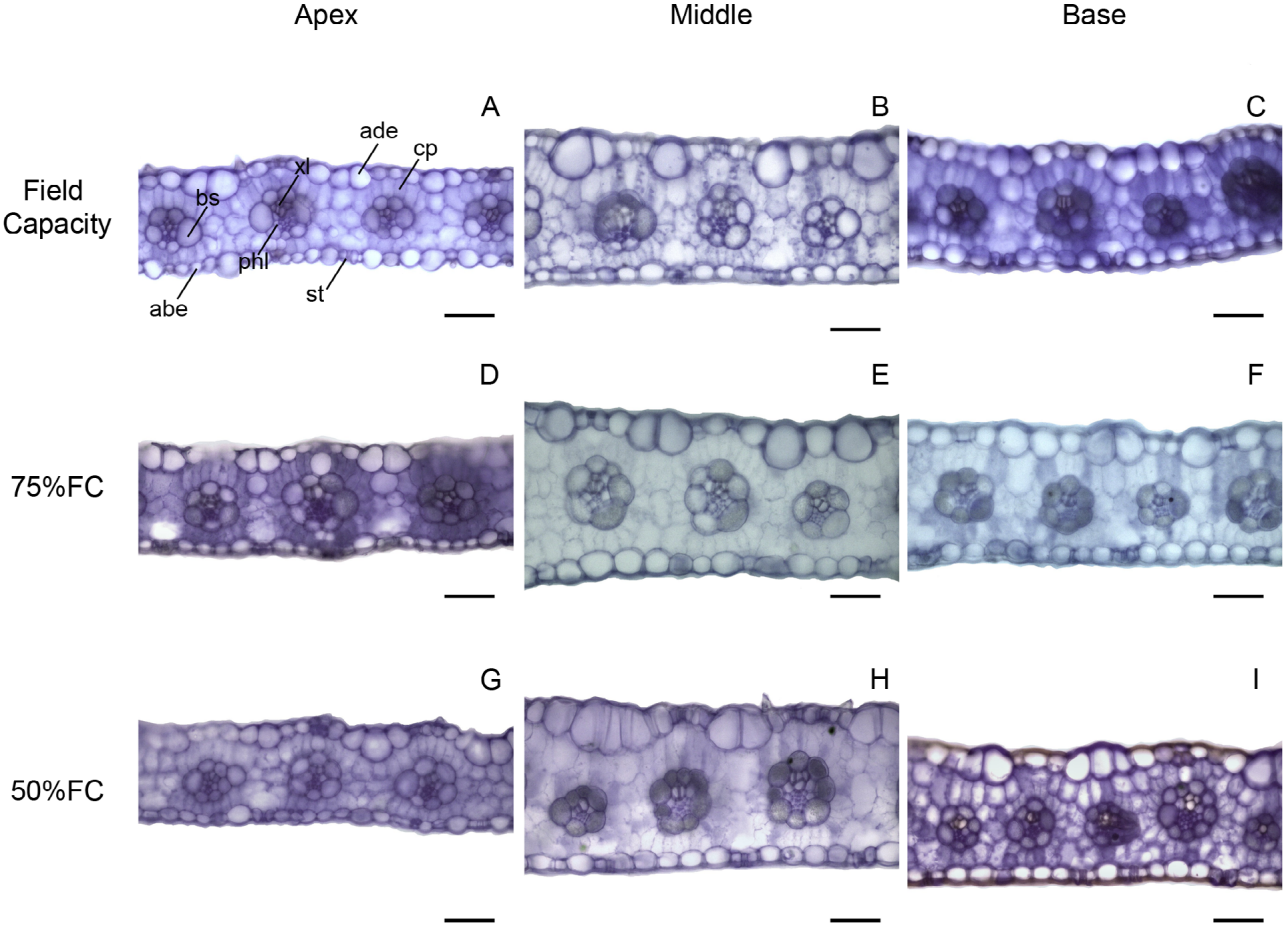
Transverse sections of *Sorghum bicolor* leaves under different water conditions. abe, abaxial epidermis; ade, adaxial epidermis; bs, bundle sheath; cp, chlorophyll parenchyma (mesophyll cells); phl, phloem; st, stomata; xl, xylem. Leaf parts: Apex = A, D, G, middle = B, E, H, Base = C, F, 1. Water treatments: Field capacity: A, B, C; 75% FC = D, E, F; 50% FC = G, H, I. Bars = 50 μm.

**Fig. 7:**
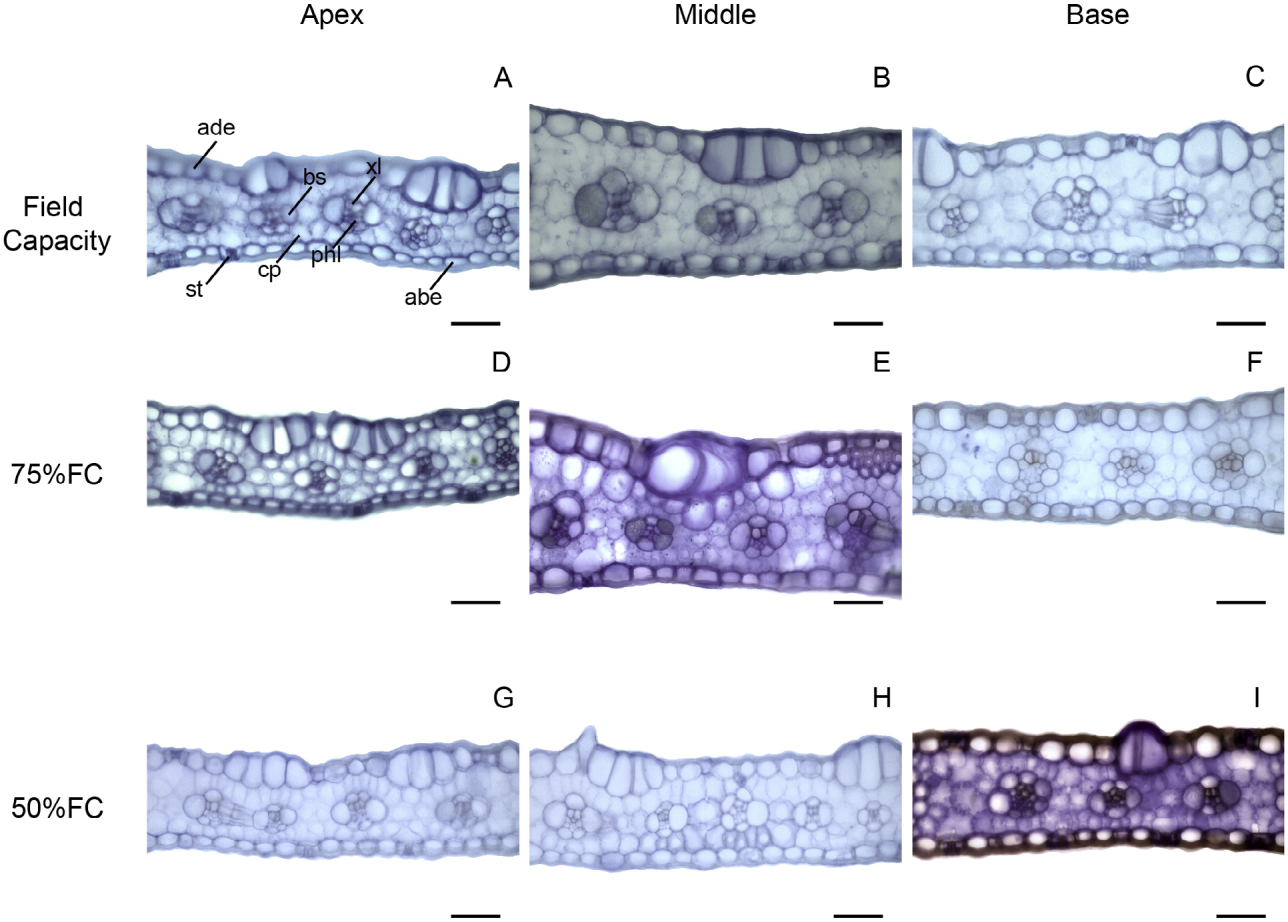
Transverse sections of *Zea mays* leaves under different water conditions. abe, abaxial epidermis; ade, adaxial epidermis; bs, bundle sheath; cp, chlorophyll parenchyma (mesophyll cells); phl, phloem; st, stomata; xl, xylem. Leaf parts: Apex = A, D, G, Middle = B, E, H, Base = C, F, 1. Water treatments: Field capacity: A, B, C, 75% FC = D, E, F, 50% FC = G, H, I. Bars = 50 μm.

Both maize and sorghum showed adequate leaf development, without severe deformations (Figs. 6 and 7) that evidence similar leaf structure under all water treatments and that maintain some degree of photosynthetic activity and growth. However, maize showed a reduction in leaf thickness and in bundle sheath under 50% FC, while sorghum showed no significant limitation to these tissues, being an important tolerance trait. Water limitation can reduce leaf thickness and chlorophyll parenchyma (Wyka *et al*. 2019; Della Torre *et al*. 2021). According to Khan *et al*. (2023), leaf thickness is related to biomass production and plant productivity because it is associated with photosynthesis. The capacity of sorghum to maintain leaf thickness and vascular bundle size is important for drought tolerance, as compared to maize.

Leaf anatomy of both maize and sorghum showed significant anatomical variations along the longitudinal axis, suggesting that middle part is more functional. Maize and sorghum both have long leaves (de Oliveira *et al*. 2023) and this morphology promotes bending of the leaf apex, which reduces light uptake in this part (Ford *et al*. 2008). It is well documented that leaf morphology influences radiation uptake (Carvalho *et al*. 2006; Dzvene *et al*. 2023). The leaf apex is thinner in both maize and sorghum leaves, with reductions in vascular bundle and xylem cell content. Smaller vascular tissue development, mainly the xylem, may be caused by environmental factors such as drought (Guha *et al*. 2018; Bijanzadeh *et al*. 2023), which may reduce vessel cavitation and favour water transport (Choat *et al*. 2016). The reduction in mesophyll and bundle sheath cells may lower photosynthetic activity because these cells are both essential for C4 photosynthesis (Wang *et al*. 2012). These results support previous works in selecting the middle part of the leaf to sample anatomical and physiological traits.

Stomata in sorghum show responses related to drought tolerance, while maize stomata showed little variation under drought. Sorghum's stomatal index and density were reduced under water limitation, which may limit water loss by transpiration, whereas in maize these traits remain unchanged or increased. Stomata control gas exchange in leaves and are important in regulating water loss and photosynthesis under drought (Cai *et al*. 2020; Zia *et al*. 2021). The reduction in number of stomata is often related to lower transpiration and reduced water loss (Gerardin *et al*. 2018; Niu *et al*. 2023). It is also important to note that most positive stomatal changes in sorghum were in the middle and basal parts of the leaves, suggesting that this leaf part is more efficient in gas exchange and control of water loss compared to the leaf apex.

Finding new tools to access drought effects and tolerance markers is essential for crop production and screening for drought‐tolerant accessions. According to Munné‐Bosch & Villadangos (2023), there is an increasing need to find tools to monitor drought stress in crops worldwide. In addition, Kreibich *et al*. (2019) suggest that the lack of data limit effective action for crop production under water limitation. In this work, the results suggest that there is a relevant modification in photosynthetic capacity and anatomical traits along the leaf longitudinal axis, and that the middle parts of the leaf of both maize and sorghum have improved traits compared to the leaf tip or base. Long leaves like those of maize may bend at the apex (Ford *et al*. 2008) and this will reduce light interception and develop less functional physiological and anatomical features. Thus, the middle part of the leaf of both maize and sorghum is more relevant when accessing functional characteristics in studies for breeding and adaptation to water limited conditions. It is important to note that, although the leaf middle part is more functional in both maize and sorghum, there is significant variation along the leaf axis in anatomical and physiological characteristics that must be considered, for instance in studies of plant shoot architecture, self‐shading and spacing, which may change the way in which radiation is intercepted by leaves. In addition, our study used one genotype for each species, and further studies must investigate other maize and sorghum material to address this limitation. Nonetheless, most studies of physiological and anatomical traits in maize and sorghum use one or few genotypes (Guha *et al*. 2018; Impa *et al*. 2019; Romanowska‐Duda *et al*. 2019; Cai *et al*. 2020; Chen *et al*. 2022; de Oliveira *et al*. 2022; de Oliveira *et al*. 2023; Dzvene *et al*. 2023). The use of one genotype for native species studies is the most common approach in non‐crop species (Hsie *et al*. 2015; Gavilanes *et al*. 2016; Mansoor *et al*. 2019; Della Torre *et al*. 2021; Lin *et al*. 2023). Therefore, results found here can be extended for other maize and sorghum genotypes but their application can be further expanded using genotypes from different regions.

## CONCLUSION

Sorghum had fewer drought‐tolerance traits compared to maize, e.g., stable photosynthesis, transpiration, chlorophyll content, and photochemical and anatomical characteristics. Most physiological traits varied along the leaf axis in both species, and the middle part of the leaf was found to be most functional, with fewer features in the apex and base. The results confirm that variations in anatomical and physiological traits in maize and sorghum leaves are related to the elongated leaf morphology.

## AUTHOR CONTRIBUTIONS

Each author contributed significantly to the final version of the work. JPVO conducted most of the experiments, data sampling, analysis, and writing of the first draft of the work. VPD, CHGR, and PNS contributed to the experiment handling, data sampling, and anatomical analysis. EMC contributed with critical review. PCM contributed with critical review. FJP was the adviser of the first author, conducted the experimental design, experiments handling data sampling, analysis, and the writing of the final version of the work.

## Supporting information


**Data S1.** ANOVA Tables.
