# Supplementary material for: Anatomical variations along the leaf axis modulate photosynthetic responses of sorghum and maize under different water availabilities
Source: Plant Biol (Stuttg). 2025 Jul 30;27(7):1366–77. doi: 10.1111/plb.70084 (PMC12631521; doi:10.1111/plb.70084)
Supplement: Supplementary file 1 — Data S1. ANOVA Tables. [file PLB-27-1366-s001.doc]

Supplementary material: ANOVA Tables

**Table 1 –** Summarized ANOVA results for all variables analyzed, including mean square values, *F* test results, and *P* values for the *Sorghum bicolor* experiment. CV% = coefficient of variation.

| ***Variable*** | ***CV %*** | ***Mean square values*** | ***F test value*** | ***P-value*** |
| --- | --- | --- | --- | --- |
| *Net photosynthesis (L) | 18.7 | 31.5 | 8.4 | 0.0015 |
| *Net photosynthesis (W) | 18.7 | 16.5 | 4.4 | 0.0220 |
| *Transpiration rate (L) | 14.6 | 0.1 | 2.8 | 0.0764 |
| *Transpiration rate (W) | 14.6 | 0.4 | 8.1 | 0.0018 |
| *Instantaneous water-use efficiency (L) | 21.6 | 46.8 | 9.9 | 0.0006 |
| *Instantaneous water-use efficiency (W) | 21.6 | 2.1 | 0.4 | 0.6403 |
| Chlorophyll content (L) | 18.2 | 49.2 | 1.1 | 0.3496 |
| Chlorophyll content (W) | 18.2 | 5.8 | 0.1 | 0.8820 |
| Actual photochemical efficiency of PSII (L) | 30.2 | 0.02 | 3.6 | 0.0317 |
| Actual photochemical efficiency of PSII (W) | 30.2 | 0.04 | 7.1 | 0.0017 |
| The maximum PSII quantum yield (L) | 3.3 | 0.002 | 1.9 | 0.1538 |
| The maximum PSII quantum yield (W) | 3.3 | 0.02 | 12.4 | <0.0001 |
| Electron transport rate (L) | 30.0 | 1031.5 | 3.0 | 0.0547 |
| Electron transport rate (W) | 30.0 | 2110.2 | 6.2 | 0.0034 |
| Leaf thickness (L) | 11.9 | 19985.9 | 38.9 | <0.0001 |
| Leaf thickness (W) | 11.9 | 1471.0 | 2.8 | 0.0621 |
| Proportion of mesophyll cells (L) | 2.2 | 36.4 | 4.0 | 0.0216 |
| Proportion of mesophyll cells (W) | 2.2 | 27.9 | 3.0 | 0.0511 |
| *Bundle sheath area (L) | 24.6 | 25784100.5 | 46.3 | <0.0001 |
| *Bundle sheath area (W) | 24.6 | 3499626.5 | 6.2 | 0.0022 |
| Proportion of the vascular tissues (L) | 33.4 | 2.2 | 0.2 | 0.7491 |
| Proportion of the vascular tissues (W) | 33.4 | 12.2 | 1.5 | 0.2104 |
| *Xylem vessel diameter (L) | 23.1 | 89.2 | 16.1 | <0.0001 |
| *Xylem vessel diameter (W) | 23.1 | 14.4 | 2.6 | 0.0756 |
| *Adaxial stomatal index (L) | 11.9 | 156.1 | 21.7 | <0.0001 |
| *Adaxial stomatal index (W) | 11.9 | 132.9 | 18.5 | <0.0001 |
| *Abaxial stomatal index (L) | 9.3 | 52.8 | 5.8 | 0.0043 |
| *Abaxial stomatal index (W) | 9.3 | 22.5 | 2.4 | 0.0893 |
| Adaxial stomatal density (L) | 13.8 | 33754.7 | 111.5 | <0.0001 |
| Adaxial stomatal density (W) | 13.8 | 2868.9 | 9.4 | 0.0002 |
| Abaxial stomatal density (L) | 13.0 | 8812.6 | 11.3 | <0.0001 |
| Abaxial stomatal density (W) | 13.0 | 721.9 | 0.9 | 0.3979 |

*The P-value* limit of the software is 0.0001, results lower than this limit are indicated as *p<*0.0001. (L) = Leaf regions (Leaf apex, leaf medium, and leaf base); (W) = Water conditions (Field capacity (FC), 75%FC and 50%FC); * = significant interaction at *p*<0.05.

**Table 2 –** Summarized ANOVA results for all variables analyzed, including mean square values, *F* test results, and *P* values for the *Zea mays* experiment. CV% = coefficient of variation.

| ***Variable*** | ***CV %*** | ***Mean square values*** | ***F test value*** | ***P-value*** |
| --- | --- | --- | --- | --- |
| *Net photosynthesis (L) | 23.7 | 32.4 | 10.6 | 0.0004 |
| *Net photosynthesis (W) | 23.7 | 2.9 | 0.9 | 0.3934 |
| *Transpiration rate (L) | 13.5 | 0.1 | 0.9 | 0.3993 |
| *Transpiration rate (W) | 13.5 | 0.2 | 14.2 | <0.0001 |
| *Instantaneous water-use efficiency (L) | 24.2 | 115.9 | 12.5 | <0.0001 |
| *Instantaneous water-use efficiency (W) | 24.2 | 10.6 | 1.1 | 0.3348 |
| Chlorophyll content (L) | 19.7 | 35.2 | 1.6 | 0.2075 |
| Chlorophyll content (W) | 19.7 | 54.3 | 2.4 | 0.0906 |
| Actual photochemical efficiency of PSII (L) | 31.3 | 0.05 | 9.8 | 0.0002 |
| Actual photochemical efficiency of PSII (W) | 31.3 | 0.01 | 2.4 | 0.0951 |
| The maximum PSII quantum yield (L) | 2.7 | 0.004 | 5.4 | 0.0067 |
| The maximum PSII quantum yield (W) | 2.7 | 0.00001 | 0.01 | 0.9882 |
| Electron transport rate (L) | 30.1 | 2628.2 | 9.2 | 0.0003 |
| Electron transport rate (W) | 30.1 | 1171.3 | 4.1 | 0.0208 |
| Leaf thickness (L) | 12.4 | 12647.4 | 29.7 | <0.0001 |
| Leaf thickness (W) | 12.4 | 2416.1 | 5.6 | 0.0048 |
| Proportion of mesophyll cells (L) | 2.2 | 4.2 | 0.4 | 0.6359 |
| Proportion of mesophyll cells (W) | 2.2 | 0.7 | 0.07 | 0.9270 |
| Bundle sheath area (L) | 35.7 | 19552146.2 | 27.3 | <0.0001 |
| Bundle sheath area (W) | 35.7 | 2347019.2 | 3.2 | 0.0389 |
| Proportion of the vascular tissues (L) | 34.0 | 24.6 | 3.8 | 0.0255 |
| Proportion of the vascular tissues (W) | 34.0 | 7.9 | 1.2 | 0.2971 |
| *Xylem vessel diameter (L) | 22.2 | 244.4 | 38.6 | <0.0001 |
| *Xylem vessel diameter (W) | 22.2 | 33.8 | 5.3 | 0.0052 |
| Adaxial stomatal index (L) | 11.6 | 6.2 | 2.1 | 0.1252 |
| Adaxial stomatal index (W) | 11.6 | 5.5 | 1.8 | 0.1578 |
| *Abaxial stomatal index (L) | 12.3 | 377.3 | 45.7 | <0.0001 |
| *Abaxial stomatal index (W) | 12.3 | 221.2 | 26.8 | <0.0001 |
| Adaxial stomatal density (L) | 12.2 | 556.6 | 4.9 | 0.0092 |
| Adaxial stomatal density (W) | 12.2 | 263.2 | 2.3 | 0.1019 |
| *Abaxial stomatal density (L) | 14.2 | 18804.2 | 37.3 | <0.0001 |
| *Abaxial stomatal density (W) | 14.2 | 4191.7 | 8.3 | 0.0005 |

*The P-value* limit of the software is 0.0001, results lower than this limit are indicated as *p<*0.0001. (L) = Leaf regions (Leaf apex, leaf medium, and leaf base); (W) = Water conditions (Field capacity (FC), 75%FC and 50%FC). * = significant interaction at *p*<0.05.
